# The effect of nano-hydroxyapatite/chitosan scaffolds on rat calvarial defects for bone regeneration

**DOI:** 10.1186/s40729-021-00327-w

**Published:** 2021-05-24

**Authors:** Emmanouil Chatzipetros, Spyros Damaskos, Konstantinos I. Tosios, Panos Christopoulos, Catherine Donta, Eleni-Marina Kalogirou, Zafeiroula Yfanti, Dimitris Tsiourvas, Aggeliki Papavasiliou, Kostas Tsiklakis

**Affiliations:** 1 Department of Oral Diagnosis and Radiology, Faculty of Dentistry, National and Kapodistrian University of Athens, 2 Thivon Str, 11527, Goudi, Athens, Greece; 2 Department of Oral Medicine and Pathology, Faculty of Dentistry, National and Kapodistrian University of Athens, Athens, Greece; 3 Department of Oral and Maxillofacial Surgery, Faculty of Dentistry, National and Kapodistrian University of Athens, Athens, Greece; 4 Institute of Nanoscience and Nanotechnology, National Centre for Scientific Research “Demokritos”, Aghia Paraskevi, Attiki, Greece

**Keywords:** Bone regeneration, Chitosan, Hydroxyapatites, Rat calvaria

## Abstract

**Background:**

This study aims at determining the biological effect of 75/25 w/w nano-hydroxyapatite/chitosan (nHAp/CS) scaffolds on bone regeneration, in terms of fraction of bone regeneration (FBR), total number of osteocytes (Ost), and osteocyte cell density (CD), as well as its biodegradability.

**Methods:**

Two critical-size defects (CSDs) were bilaterally trephined in the parietal bone of 36 adult Sprague-Dawley rats (18 males and 18 females); the left remained empty (group A), while the right CSD was filled with nHAp/CS scaffold (group B). Two female rats died postoperatively. Twelve, 11, and 11 rats were euthanized at 2, 4, and 8 weeks post-surgery, respectively. Subsequently, 34 specimens were resected containing both CSDs. Histological and histomorphometric analyses were performed to determine the FBR, calculated as [the sum of areas of newly formed bone in lateral and central regions of interest (ROIs)]/area of the original defect, as well as the Ost and the CD (Ost/mm^2^) in each ROI of both groups (A and B). Moreover, biodegradability of the nHAp/CS scaffolds was estimated via the surface area of the biomaterial (BmA) in the 2nd, 4th, and 8th week post-surgery.

**Results:**

The FBR of group B increased significantly from 2nd to 8th week compared to group A (*P* = 0.009). Both the mean CD and the mean Ost values of group B increased compared to group A (*P* = 0.004 and *P* < 0.05 respectively). Moreover, the mean value of BmA decreased from 2nd to 8th week (*P* = 0.001).

**Conclusions:**

Based on histological and histomorphometric results, we support that 75/25 w/w nHAp/CS scaffolds provide an effective space for new bone formation.

## Background

The autologous bone graft is the gold standard augmentation material due to its osteoconductive, osteoinductive, and osteogenic properties [1], but complications and/or insufficient bone quantity and/or quality at the donor site may limit its utilization [2]. Therefore, numerous bone graft materials, allografts and/or xenografts, were created in order to supply an osteoconductive matrix and enhance bone formation in the so-called hard tissue critical-size defect (CSD) [3], i.e., the smallest in diameter bone defect that does not heal spontaneously [4]. However, the use of those materials runs the risks of disease transmission, infection, resorption, and immune rejection [5].

An ideal bone graft material should have properties, such as biocompatibility, osteoinductivity, osteoconductivity, controlled biodegradability, and ability to deliver cells, support differentiation of regenerative cells, and promote growth of new bone into the defect’s area [6]. Hence, composite frameworks or scaffolds for bone regeneration should possess properties in a level close to those of bone tissue [7], also having a controlled pore structure that would allow cells proliferation, migration, and growth [8].

Present research is directed towards the application of porous, three-dimensional scaffolds composed of both inorganic and organic, mainly polymeric, constituents that should be non-immunogenic, osteoconductive, biocompatible, and hemocompatible. Among a variety of biopolymers that have been employed to this end, such as gelatin, collagen, proteoglycans, and alginates [9, 10], chitosan (CS) or its derivatives is characterized for its antibacterial properties, biocompatibility, and the ability to form porous structures with a level of porosity that is appropriate for cell ingrowth and osteoconduction [11]. CS is also structurally similar with the glycosaminoglycans that are present in extracellular matrices and play a significant role in modulating the morphology, differentiation, and function of chondrocytes [12].

In biomedical applications, CS scaffolds incorporate bioceramics, such as synthetic hydroxyapatite (HAp) that, apart from improving the mechanical properties of the CS-based bone graft [13], is an excellent bone substitute due to its biocompatibility, non-toxicity, non-immunogenic behavior, and osteoconductive ability [7, 13]. Porous scaffolds, usually consisting of nano-Hydroxyapatite (nHAp) and CS (nHAp/CS), have been used in bone regeneration [14–17], showing improved pre-osteoblasts response, high cell attachment, and proliferation, as well as well-spread cell distribution within the structure of biomaterial [15]. In order to create a nHAp/CS scaffold with mechanical properties close to normal bone, various concentrations of nHAp have been tested [8, 14]. Given that the presence of nHAp enhances the mechanical properties of the scaffolds, the highest possible nHAp content is desirable to attain mechanical properties as close as those of the bone. Kashiwazaki et al. [14] reported that 80/20 w/w of nHAp/CS scaffolds showed, after heat treatment with saturated steam, enhanced mechanical strength, as well as good biocompatibility and biodegradability. Tsiourvas et al. [8] observed in vitro that when the nHAp concentration exceeds 80% the resulting scaffolds were friable and suggested that 75/25 w/w of nHAp/CS could provide scaffolds with improved physicomechanical properties.

We have initially studied the mechanical properties of 75/25 w/w nHAp/CS scaffolds [8]. Next, in a pilot *in vivo* study, we used histological and histomorphometric analysis on 6 rat calvaria to document the biological behavior of 75/25 w/w of nHAp/CS biomaterial via assessment of the area of new bone formation (NBF), the total number of osteocytes included in it and the evaluation of any material-associated inflammatory reaction [16]. Areas of NBF were histologically confirmed in additional 28 rat calvaria in a subsequent study that focused on the cone beam computed tomography (CBCT) imaging characteristics of 75/25 w/w nHAp/CS [17]. Herein, we aim to further elaborate on and expand our previous research by an extended histomorphometric approach, in order to better delineate the biological effect of 75/25 w/w nHAp/CS scaffolds on bone regeneration, by examining the fraction of bone regeneration (FBR), the total number (Ost), and the cell density (CD; Ost/mm^2^) of osteocytes in areas of NBF, as well as the biodegradability of nHAp/CS scaffolds.

## Methods

### Subjects

The required sample size was determined by power analysis (Power 1-β err prob = 0.6949) using one-way ANOVA Fixed effects (IBM SPSS 25.0, IBM Corp., Armonk, NY, USA), according to the aims of “Animal Research: Reporting of in vivo Experimental guidelines (ARRIVE)” [18], resulting in 36 Sprague-Dawley adult rats, 18 males and 18 females. These were approximately 3-month-old and weighed more than 250 gr. Of these, 6 were used in our previous pilot study [16], and 30 in our previous CBCT study (prior to the decalcification process) [17]. Thus, the required number of experimental animals was minimized, in accordance with the ARRIVE guidelines [18]. All animal handling and surgical procedures were conducted in accordance with the guidelines of animal care and the use of laboratory experimental animals. For the aims of the present study, three study groups were created for 2, 4, and 8 weeks post-surgery. Each group was comprised of 12 rats (6 males and 6 females). The study was approved by the Directorate of Agricultural and Veterinary Policy (protocol number 1181/2-03-2017 and registration code EL 25 BIO 05, Athens, Greece). The study protocol was in line with EU Directive 2010/63/EU, based on the concept of replacement, reduction, and refinement of animal studies (the 3R principle).

### Preparation and characterization of nano-hydroxyapatite/chitosan scaffolds

**Fig. 1:**
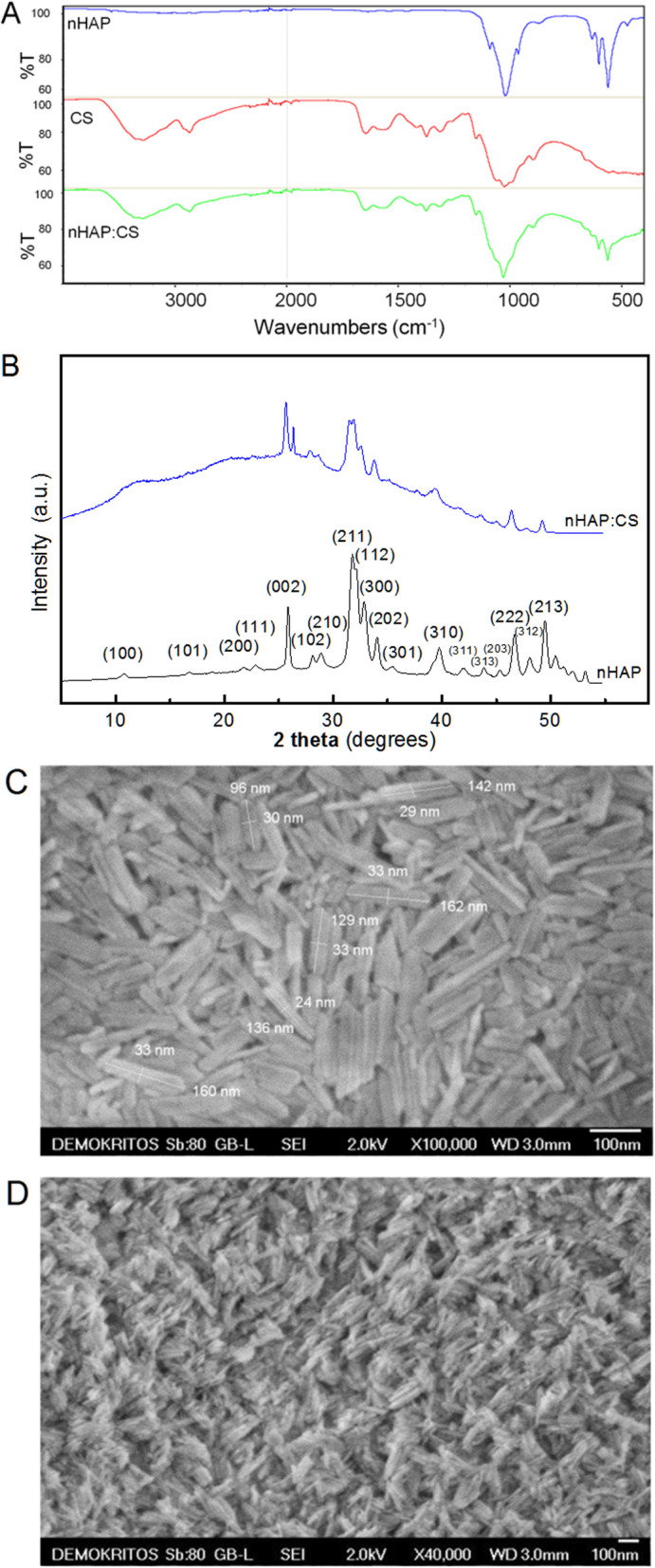
Fourier transform infrared (FTIR) spectra of synthesized hydroxyapatite nanoparticles (nHAp), chitosan (CS), and nHAp/CS scaffolds (**a**). X-ray diffraction patterns of nHAp and nHAp/CS scaffolds (**b**). High resolution SEM images of nHAp in powder form (**c**) or embedded in the chitosan matrix of nHAp/CS scaffolds (**d**)

Nanoparticles of hydroxyapatite (nHAp) were synthesized according to our previous work [19] in the presence of hyperbranched polyethylene imine (Lupasol G100, BASF, Greece), employed to control the size and morphology of hydroxyapatite crystals. Both the attenuated total reflectance-Fourier transform infrared (ATR-FTIR) spectrum (Nicolet 6700 spectrometer, Thermo Scientific, Waltham, MA, USA) that shows the typical peaks of HAp [19] (Fig. 1a) and the X-ray powder diffractogram (Rigaku rotating anode X-ray generator, coupled with an R-AXIS IV image plate, Rigaku Co., Tokyo, Japan) that shows excellent agreement and presents the characteristic diffraction pattern of hexagonal pure HAp (JCPDS 9-432) (Fig. 1b), proved the crystalline structure and phase purity of the synthesized HAp. In addition, scanning electron microscope (SEM) images (JSM 7401F, JEOL Ltd., Tokyo, Japan) revealed that the obtained nanoparticles were monodisperse rod-like crystals with diameters of 20–40 nm and lengths of circa 80–160 nm (Fig. 1c). Furthermore, they were able to be perfectly dispersed in water at concentrations up to 9wt%, a property that has remained stable for more than a year.

**Fig. 2:**
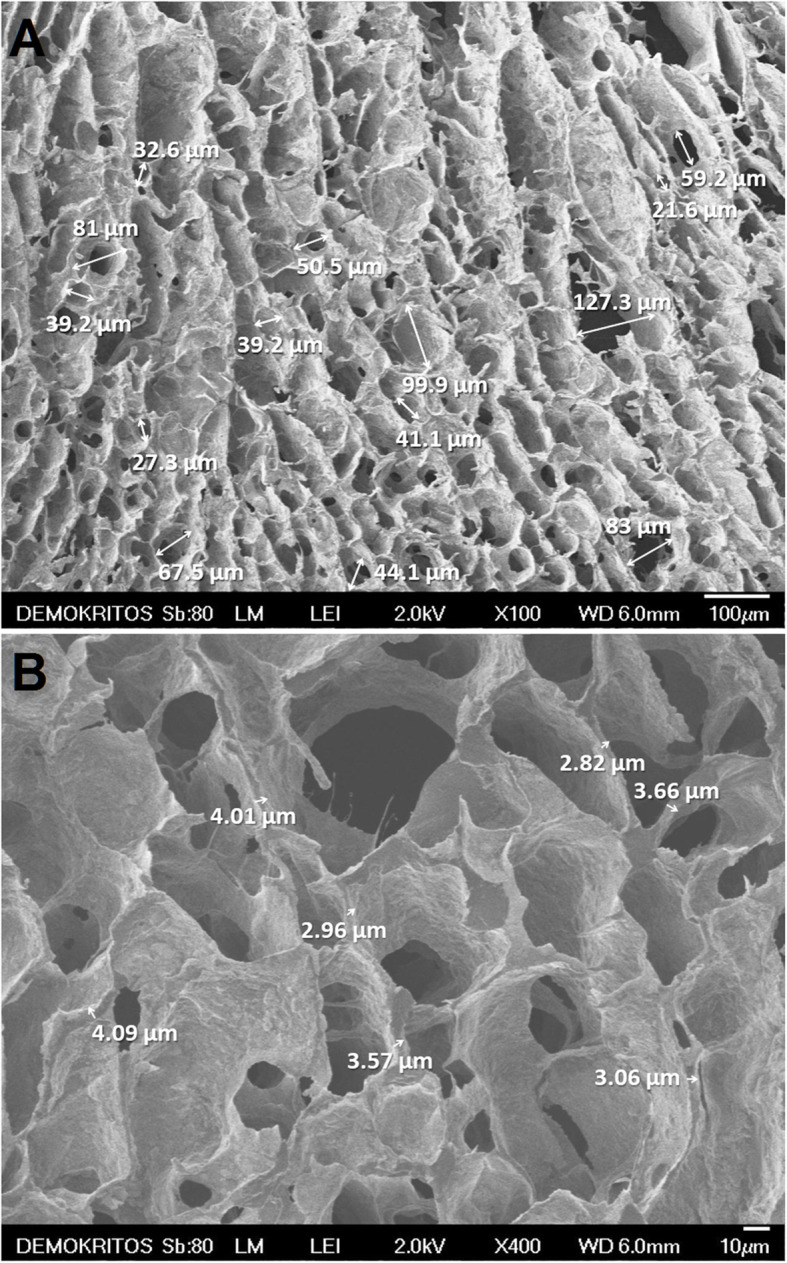
SEM images of the scaffolds indicating the pore shape and size (**a**), and the pore wall thickness (**b**)

Composite porous scaffolds were developed [8] by preparing a 3%w/w CS (Aldrich, high-molecular weight, deacetylation degree ≥ 75%) solution in aqueous acetic acid (1.5%w/w) and adding nHAp to a final HAp:CS weight ratio of 75:25. The resulting thick slurry was thoroughly mixed and molded in glass tubes (5 mm i.d.) that were subsequently frozen at – 25 ^o^C and lyophilized. The derived HAp:CS cylindrical porous scaffolds (5 mm diameter) were cut to disks 1 mm thick, ethanol sterilized, and extensively washed with sterile phosphate buffer saline inside a laminar flow cabinet. ATR-FTIR spectroscopy of the scaffolds confirmed the presence of HAp and CS (Fig. 1a), while the X-ray diffractogram also revealed crystalline nHAp and of amorphous, due to the existence of very broad peaks at about 10 and 20° CS. The dispersibility of nHAp in the CS matrix, as well as the morphology, size, and shape of the pores were investigated using a field emission scanning electron microscope (JSM 7401F, JEOL Ltd., Tokyo, Japan) equipped with Gentle Beam mode. High-resolution SEM images of scaffolds revealed fine dispersion of the rod-like elongated HAp crystals in the CS matrix (Fig. 1d) and the absence of agglomerates. Lower magnification SEM images of the porous structure of nHAp/CS scaffold (Fig. 2a) revealed the presence of pores less than 150 nm, typically 20–100 nm and also allowed the wall thickness of the HAp/CS to be determined about 3–4 μm (Fig. 2b). Finally, the porosity and total pore volume were found to be 85 ± 1% and 5.0 ± 0.5mL/g, respectively, as established by determining the volume of liquid infused in the pores of dried scaffolds [20].

### Surgical procedure

**Fig. 3:**
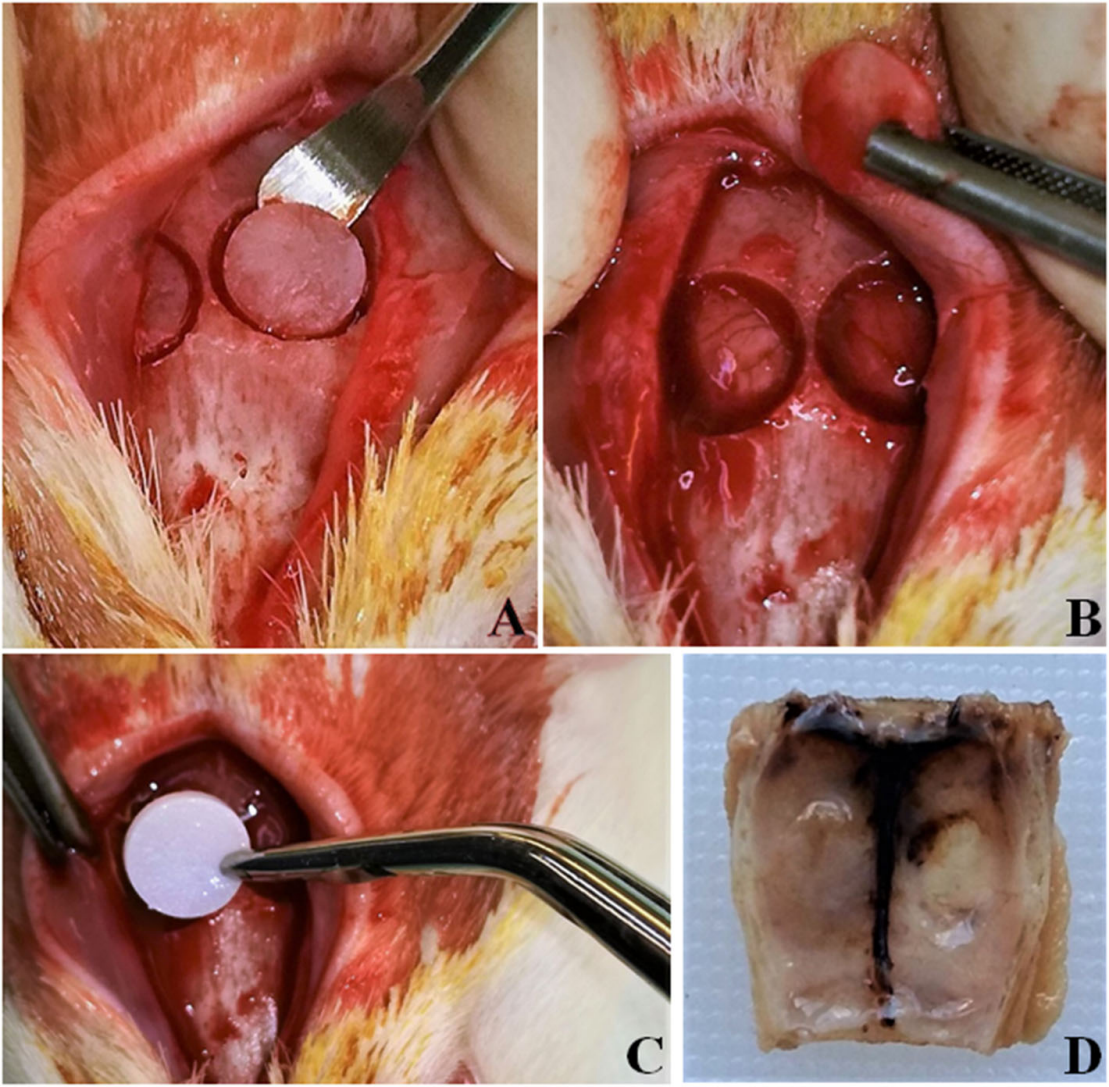
Two–5 mm in diameter—full thickness critical size defects were trephined in both sides of the parietal bone (**a, b**). A 75/25 w/w nHAp/CS scaffold was placed in the right defect, while the left remained empty (**c**). The excised specimen (15 × 2 × 10 mm) of rat’s calvaria (**d**)

Preoperatively, each animal was given a thorough clinical examination and complete hematological/biochemical tests. General anesthesia was given by intramuscular injection with xylazine 5 mg/kg (Rompun, Bayer Animal Health GmbH D-51368, Leverkusen, Germany) and ketamine hydrochloride 100 mg/kg (IMALGENE 1000, MERIAL, 29 Avenue Tony Garnier, 69007 Lyon, France). After shaving and painting with povidone-iodine (Betadine Solution, Lavipharm, Athens, Greece), a 2-cm longitudinal midsagittal cutaneous incision was made on the scalp. The musculature and the periosteum were exposed under the skin to allow for the periosteal dissection procedure [21]. Subsequently, two symmetrical round bone CSD were created in the dorsal part of the right and the left parietal bones (Fig. 3a), using a dental trephine burr of 5 mm diameter (MT-00500, MIS, Israel) operated at 10,000 rpm under sterile saline irrigation (Sodium Chloride 0.9% Intravenous Infusion, BIOSER, Greece). This diameter was chosen for allowing proper fitting of a nHAp/CS scaffold that was 5 mm in diameter and 1 mm thick. The whole procedure was done with caution to avoid damage to the dura mater or the superior sagittal sinus, and the engagement of the midsagittal suture and periosteum [22, 23]. Τhe native periosteum is of great biological importance, as it may act as a source of osteoprogenitor elements and contributes to graft osseointegration [23]. The CSD on the left parietal bone was left empty of biomaterial (group A: control group), while the CSD on the right parietal bone was loaded with a scaffold 75/25 w/w nHAp/CS (group B: experimental group) (Fig. 3b, c). The wound was sutured in layers. The periosteal flap was reflected over the defects and sutured to the contralateral side using 4–0 polyglycolic acid suture (PGA 4–0, medipac, Greece). The skin was then closed using 3–0 polyglactin 910 sutures (Coated VICRYL, Ethicon, Johnson&Johnson, USA). The postoperative stage included antimicrobial treatment by intramuscular injection with enrofloxacin 2.5 mg/kg (Baytril 5%, Bayer Animal Health GmbH D-51368 Leverkusen, Germany), as well as analgesic and anti-inflammatory treatment with carprofen (Rimadyl, Pfizer, USA).

Two female rats died postoperatively, while the remaining 18 male and 16 female animals were euthanized with diethyl ether (Sigma–Aldrich, USA) inhalation as follows: 12 (6 males, 6 females) at 2 weeks, 11 (6 males, 5 females) at 4 weeks, and 11 (6 males, 5 females) at 8 weeks post-surgery. The rats’ calvaria were properly cut off and excised using a surgical sawmill. The 34 specimens included both the parietal bones and parts of occipital and frontal bones. The dimensions of each specimen were approximately 15 mm wide, 2 mm thick, and 10 mm long (15 × 2 × 10 mm) (Fig. 3d).

### Histological analysis

**Fig. 4:**
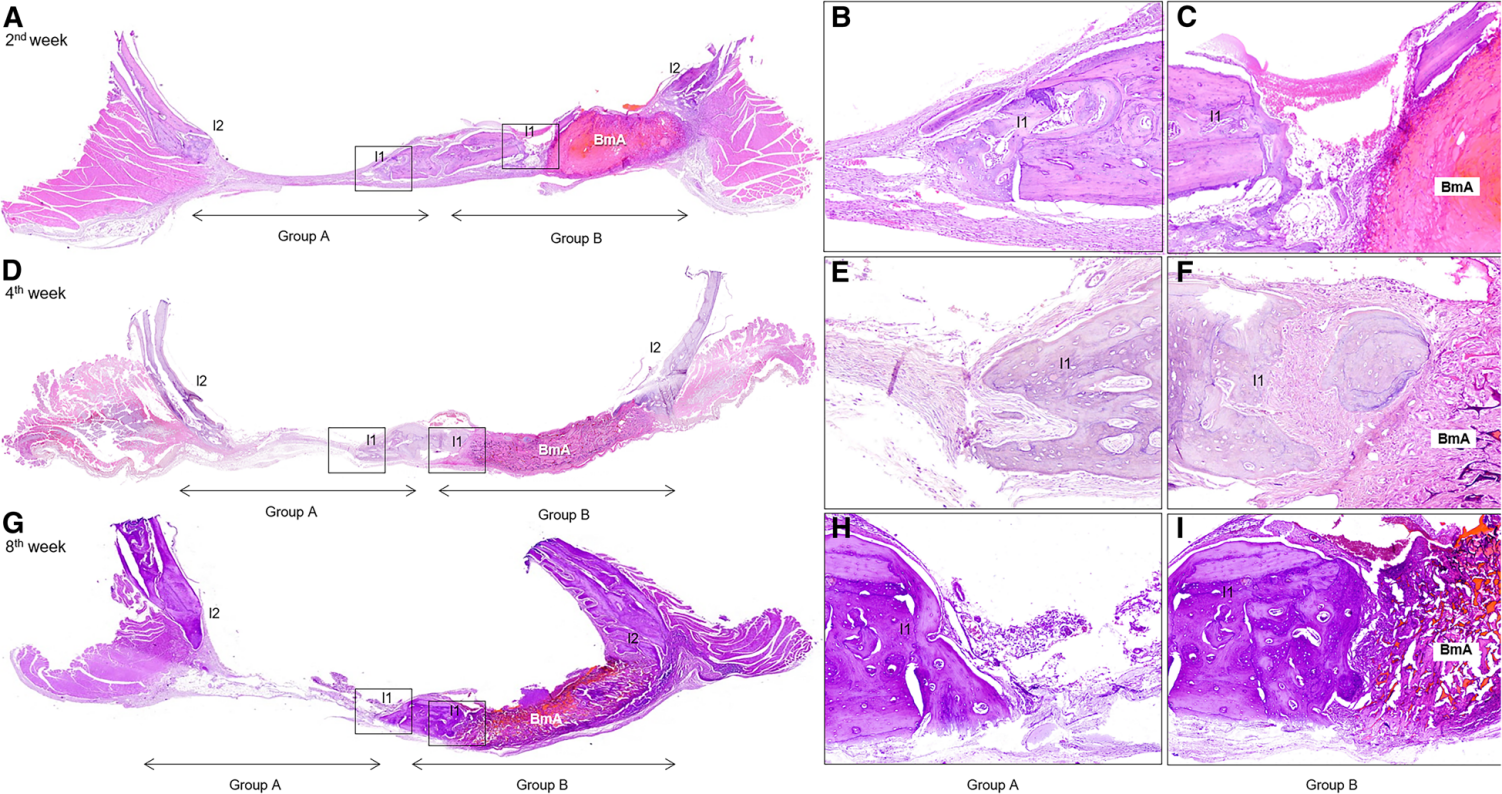
New bone formation (NBF) over time in both control and experimental groups; in rats euthanized at 2 weeks postoperatively (**a–c**), some NBF was observed on both experimental and control group, but not in the central area. At 4 weeks (**d–f**) and 8 weeks (**g–i**) postoperatively, a greater NBF area was observed on the inward (lateral 1; l_1_) or outward (lateral 2; l_2_) of middle sagittal seam lateral and in some samples also on the central region of the experimental group relative to the control group. The surface area of the biomaterial (BmA) decreased between the 2nd and 8th week post-surgery [hematoxylin and eosin stain, original magnifications for **a**, **d** and **g** × 20. Inset figures **b**, **c**, **e**, **f, h**, and **i** were prepared using ObjectiveViewer (https://www.objectivepathology.com/objectiveview)]

The 34 bone specimens were immediately fixed in 10% neutral buffered formalin solution for 24 h, decalcified in an EDTA-based solution (MicroDec, Diapath, Italy) for 7 days, and embedded in paraffin. Ten transverse 5-μm-thick tissue sections were prepared from each specimen utilizing the standard histological technique and stained with hematoxylin-eosin solution. Tissue section included the mid-point of both CSDs in the coronal plane, and those providing a technically sufficient view of both CSDs (A and B) was further evaluated (Fig. 4).

### Histomorphometric analysis

**Fig. 5:**
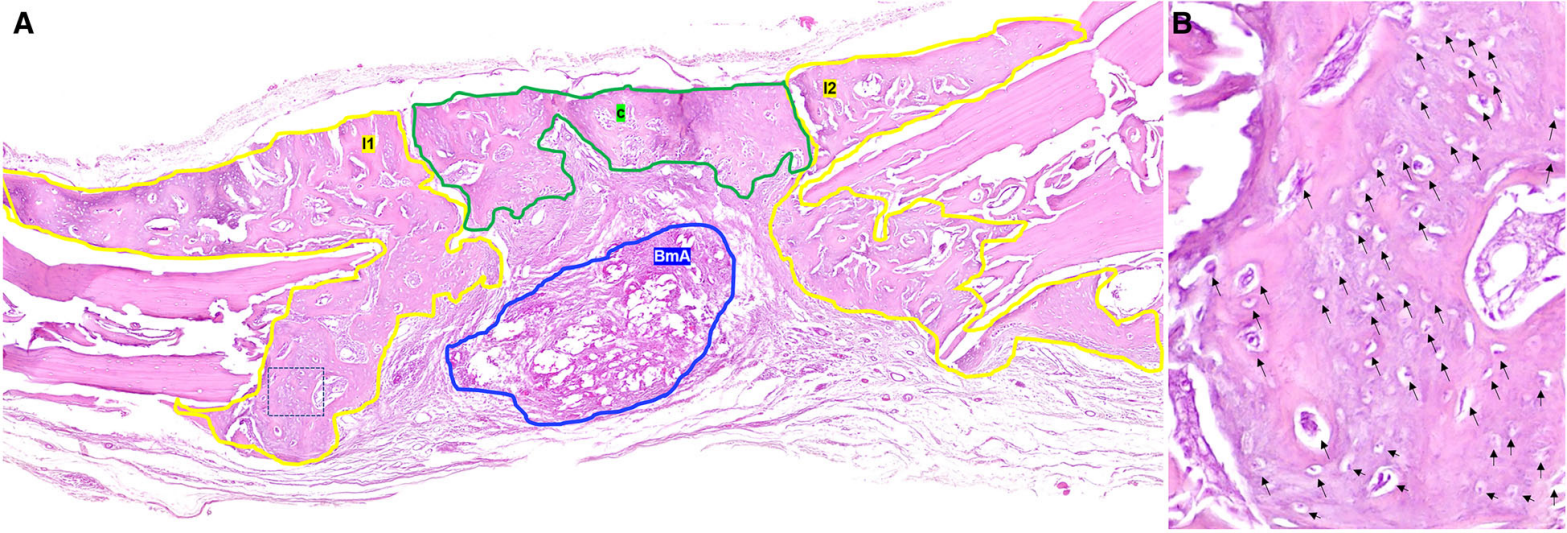
Using a digital drawing and crop tool the regions of interest (ROIs) were segmented relative to the adjacent structures (**a**). The *yellow line* designates the lateral area inward (l_1_) or outward (l_2_) of middle sagittal seam of the newly formed bone and the *green line* designates the area of central bone formation (**c**), while the *blue circular line* specifies the area of the remained biomaterial (BmA). Τhe total number of osteocytes (Ost**-***arrows*) in the newly formed bone surface was identified and used to estimate the osteocyte cell density (CD; Ost/mm^2^) in each ROI of both experimental and control groups (**b**) [hematoxylin and eosin stain, original magnifications for A × 20. Inset figure **b** was prepared using ObjectiveViewer (https://www.objectivepathology.com/objectiveview)]

This was carried out by an oral pathologist to whom the time of intervention and euthanasia was not disclosed (concealment) [24]. The digital image analysis software used for histomorphometric analysis was Image Pro-Plus v6.0.0.260 (Media Cybernetics, Rockville, MD, USA). Histomorphometric analysis focused on bone regeneration, assessed in terms of FBR [21], total osteocyte number (Ost) and osteocyte cell density (CD) [25], and on biodegradability of the nHAp/CS scaffolds. The NBF areas (expressed in μm^2^), used to calculate the FBR and CD, were traced using a digital drawing and cropping tool (Creative Pen Tablet WACOM; Wacom Europe GmbH, Germany). The regions of interest (ROIs) were segmented relative to the adjacent structures as follows: (a) lateral area inward of middle sagittal seam (lateral 1; l_1_), (b) lateral area outward of middle sagittal seam (lateral 2; l_2_), (c) central area (central; c), and (d) area of the original defect (od), that corresponded to the histological profile of the CSD (Fig. 5a). The FBR was calculated as follows: FBR = (l_1_ + l_2_ + c)/od × 100%. In addition, the Ost and the CD (Ost/mm^2^) for each ROI of both groups (A and B) were measured (Fig. 5b). Biodegradability of the nHAp/CS scaffolds was estimated by comparing the surface area occupied by the CS scaffold (biomaterial’s area, BmA) in the 2nd, 4th, and 8th week post-surgery [23, 26] (Fig. 4a, d, g; Fig. 5a).

### Statistical analysis

All measurements of the 34 specimens [surface as μm^2^ (l_1_, l_2_, c, od, BmA), Ost, FBR, CD] were estimated as mean ± standard deviation (SD). One-way analysis of variance (ANOVA), *t* test, and post hoc tests (Bonferroni) were used for statistical analysis. All data were analyzed using IBM SPSS 25.0 (Chicago, IL, USA). The significance level was set to *P* < 0.05.

## Results

### Histological results

Sixty-eight CSDs were available for microscopic evaluation (34 for each group; A and B). NBF appeared as fibrous bone with numerous osteocytes that could be easily distinguished from the pre-existing calvarial bone at the edges of the CSDs that was lamellar and less cellular, and nHAp/CS as a cellular, amorphous eosinophilic material, circumscribed by vascular and cellular fibrous connective tissue (Fig. 4).

In the 2nd postoperative week NBF was observed on the l_1_ and l_2_ ROIs of both CSD groups, but not in the c ROI (Fig. 4a–c). nHAp/CS occupied most of the CSD and was surrounded by a few foreign body multinucleated giant cells and a mild inflammatory infiltration, consisting mostly of lymphocytes and plasmacytes. In the 4th (Fig. 4d–f) and 8th (Fig. 4g–i) postoperative week, NBF was observed on the l_1_ and l_2_ ROIs of group B that was almost equally distributed, while NBF was observed in the c ROI of 13 rats. nHAp/CS appeared mostly fragmented, with intervening fascicles of fibrous connective tissue, while inflammatory cells were sparse, and no multinucleated giant cells were seen. Necrosis or pus formation was not seen in any of the CSD of the group A and B.

### Histomorphometric results

No significant difference in FBR was found between male and female rats (*P* = 0.06). In contrast, when samples of all weeks were analyzed together in each group, the mean value of FBR in group B (33.84%) was significantly higher than that in group A (15.92%) (*P* = 0.000 < < 0.001) (Table 1). In addition, the mean value of FBR increased significantly from the 2nd to the 8th week in group B compared to group A (*P* = 0.009) (Table 2).

**Table 1:** The experimental group (group B) showed a statistically significant increase in the mean value of fraction of bone regeneration (%) (FBR_B = 33.84%) relative to the control group (group A) (FBR_A = 15.92%) (*P* = 0.000 < < 0.001). Group B also showed a statistically significant increase in the mean value of cell density (CD; osteocytes/mm^2^) (CD_B = 519.56 Ost/mm^2^) relative to the group A (CD_A = 414.48 Ost/mm^2^) (*P* = 0.004).

Parameters	Mean	*N*	Std. deviation	Std. error mean	*P* value
^a^FBR_A	15.92	34	7.32	1.26	0.000 < < 0.001
^b^FBR_B	33.84	34	18.91	3.24	
^c^CD_A	414.48	34	117.79	20.20	0.004
^d^CD_B	519.56	34	197.89	33.93	

^a^FBR_A = mean value of fraction of bone regeneration (%) for group A (control group)

^b^FBR_B = mean value of fraction of bone regeneration (%) for group B (experimental group)

^c^CD_A = mean value of cell density (osteocytes/mm^2^; Ost/mm^2^) for group A (control group)

^d^CD_B = mean value of cell density (osteocytes/mm^2^; Ost/mm^2^) for group B (experimental group)

**Table 2:** The mean value of fraction of bone regeneration (FBR) of the experimental group (group B; FBR_B) increases from the 2nd to the 8th week compared to FBR of the control group (group A; FBR_A) (*P* = 0.009). More in detail, at the 2nd postoperatively week the FBR of group A was 14.88%, while the FBR of group B was 19.96%. Four weeks postoperatively, the FBR of group A was 16.73%, while the group’s B FBR was 39.13%. Finally, 8 weeks postoperatively the FBR of group A was 15.98%, while the FBR of group B was 42.13%

Group	Week	Mean	Std. error	95% confidence		
				Lower bound	Upper bound	*P* value
^a^FBR_A	2	14.88	3.45	7.96	21.80	0.009
	4	16.73	3.62	9.47	23.98	
	8	15.98	3.62	8.72	23.23	
^b^FBR_B	2	19.96	3.45	13.04	26.87	
	4	39.13	3.62	31.88	46.39	
	8	42.13	3.62	34.87	49.38	

^a^FBR_A = mean value of fraction of bone regeneration (%) for group A (control group)

^b^FBR_B = mean value of fraction of bone regeneration (%) for group B (experimental group)

Group B showed a significant increase in the mean value of Ost relative to group A in all ROIs: 1_1_ (*P* = 0.000 < < 0.001), 1_2_ (*P* = 0.000 < < 0.001), and c (*P* = 0.040) (Table 3), while the mean value of Ost increased significantly from 2nd (340.00 Ost) to 8th week (661.18 Ost) in the 1_2_ region of group B (*P* = 0.025) (Table 4).

**Table 3:** The experimental group showed a statistically significant increase in mean value of the total number osteocytes (Ost) relative to the control group in all regions of interest (ROIs: l1, l2, and central): 1ateral 1: B_Ost_l1 = 247.70 osteocytes, A_Ost_l1= 87.88 osteocytes (*P* = 0.000 < < 0.001) 1ateral 2: B_Ost_l2 = 465.82 osteocytes, A_Ost_l2 = 165.97 osteocytes (*P* = 0.000 < < 0.001) Central: B_Ost_Central = 39.32 osteocytes, A_Ost_Central = 10.20 osteocytes (*P* = 0.040)

		Mean	***N***	Std. deviation	Std. error mean	***P*** value
	^a^**A_Ost_l**_**1**_	87.88	34	60.93	10.45	<< 0.001
	^b^**B_Ost_l**_**1**_	247.70	34	215.04	36.87	
	^a^**A_Ost_l**_**2**_	165.97	34	77.52	13.29	<< 0.001
	^b^**B_Ost_l**_**2**_	465.82	34	299.39	51.34	
	^a^**A_Ost_Central**	10.20	34	24.92	4.27	0.040
	^b^**B_Ost_Central**	39.32	34	70.57	12.10	

^a^: A_Ost = mean value of osteocytes of control group

^b^: B_Ost = mean value of osteocytes of experimental group

**Table 4:** The mean value of osteocytes (Ost) increased significantly from 2nd (340.00 Ost) to 8th week (661.18 Ost) in the 1_2_ region of group B (*P* = 0.025)

Group	Week	Mean	Std. deviation	Std. error	
					*P* value
^a^A_Ost_l_2_	2	173.08	67.27	19.42	2→4 week: 0.936 2→8 week: 1.000
	4	139.81	55.00	16.58	2→4 week: 0.936 4→8 week: 0.563
	8	184.36	103.16	31.10	2→8 week: 1.000 4→8 week: 0.563
^b^B_Ost_l_2_	2	340.00	166.44	48.04	2→4 week: 1.000 2→8 week: 0.025
	4	407.72	118.53	35.74	2→4 week: 1.000 4→8 week: 0.112
	8	661.18	431.95	130.24	2→8 week: 0.025 4→8 week: 0.112

^a^A_Ost_l_2_ = mean value of osteocytes of control group in lateral 2 region of interest

^b^B_Ost_l_2_ = mean value of osteocytes of experimental group in lateral 2 region of interest

There was a significant increase in the mean value of CD in group B (519.56 Ost/mm^2^) compared to group A (414.48 Ost/mm^2^) (*P* = 0.004) (Table 1).

Regarding biodegradability, no significant difference was observed in the mean value of the BmA between male and female rats (*P* = 0.330). In contrast, in group B a significant reduction was found from 2nd (3545719.83 μm^2^) to 8th week (1907642.54 μm^2^) (*P* = 0.001) (Table 5).

**Table 5:** The mean value of the surface area (in μm^2^) of the residual biomaterial (BmA) of nano-hydroxyapatite/chitosan (nHAp/CS) scaffold was reduced from 2nd (3545719.83 μm^2^) to 4th week (1114298.18 μm^2^) (*P* = 0.000 < < 0.001), as well as from 2nd (3545719.83 μm^2^) to 8th week (1907642.54 μm^2^) (*P* = 0.001). The apparent increase in BmA from the 4th (1114298.18 μm^2^) to 8th week (1907642.54 μm^2^) was not statistically significant (*P* = 0.204)

	Week	Mean	Std. deviation	Std. error	95% confidence		
					Lower bound	Upper bound	*P* value
^a^BmA	2	3545719.83	1339540.27	386691.96	2694616.55	4396823.11	2→4 week: 0.000 2→8 week: 0.001
	4	1114298.18	575732.74	173589.95	727515.66	1501080.70	2→4 week: 0.000 4→8 week: 0.204
	8	1907642.54	836147.56	252107.97	1345910.96	2469374.12	2→8 week: 0.001 4→8 week: 0.204

^a^*BmA* = mean value of surface area (in μm^2^) of the residual biomaterial (nano-hydoxyapatite/chitosan scaffold, nHAp/CS)

## Discussion

The present study documents both histologically and histomorphometrically that the 75/25 w/w nHAp/CS scaffolds have a positive effect on bone regeneration in rats’ calvarial 5-mm-diameter CSD, as is estimated by FBR, Ost, CD, and biodegradability.

CSD is the smallest in diameter bone defect that does not heal spontaneously [4], but its proper dimensions is a matter of dispute, as most researchers recommend a 5-mm-diameter CSD [16, 17, 22, 23, 27–30] and others an 8-mm diameter [21, 31, 32]. In our study, the 5-mm-diameter CSD in group A did not heal throughout the experiment, as the NBF remained stable in all weeks (2nd, 4th, and 8th). In addition, it allowed the simultaneous creation of two-sided CSDs for the comparative evaluation of the experimental and control groups in the same animal, resulted in reduced morbidity and mortality of rats, and allowed the use of less experimental animals [23, 33].

Histomorphometric analysis is considered the “gold standard” for the evaluation of NBF in rats’ calvarial CSDs [16, 21, 23, 29, 34] and is assessed by FBR [21], Ost count [16], and CD [25]. In the present study, the use of a digital image analysis system (Image Pro-Plus v6.0.0.260) allowed the objective comparison of NBF among the CSDs of both groups (A and B). It was found an increase in group B at all weeks (2nd, 4th, and 8th) compared to group A for FBR, Ost count and CD, suggesting that the 75/25 w/w nHAp/CS biomaterial advance the bone regeneration process by promoting NBF over time.

The results of our study are consistent with those of previous experimental studies [21, 30, 35, 36]. In particular, Kim et al. [21] showed that at 4 weeks postoperatively the mean FBR value of the control group was 30.50%, the Bio-Oss group 28.53%, and the HAp group 42.90%. In the same postoperative week, the FBR of our study for group A was 16.73% and 39.13% for group B, respectively. At 8 weeks postoperatively, Kim et al. [21] showed that the mean FBR value of the control group was 50.21%, the Bio-Oss group 54.12% and the HAp group 50.92%, respectively, while, in our study, the FBR for group A was 15.98%, and 42.13% for group B, respectively. However, in that study [21], the authors found that both the control and experimental groups showed progressive healing throughout the experiment, although an 8-mm CSD has been selected. Any difference between our results and those of Kim et al. [21], regarding the healing rate in the control group, can be attributed to the small sample size of that study, [10 in the study of Kim et al. [21] versus 34 in our study]. It is worth noting that in our study, NBF was observed in the central area of group B in 13 rats. This probably indicates the tendency of biomaterial to fill the CSD, presenting a centripetal new bone formation.

Biodegradability of a scaffold graft biomaterial is a key element of the bone regeneration pathway [23], as it leads to gradual absorption of the implanted biomaterial and its replacement by bone tissue. The biological mechanism of biodegradability involves a local inflammatory reaction, where the release of free radicals and peroxide anions, and synergy with angiogenesis and osteogenesis promotes the local action of macrophages [35, 37]. Biodegradability of the 75/25 w/w nHAp/CS scaffolds in our study was assessed by studying at various time intervals the residual CS, i.e., the organic component of the biomaterial, given that the second component, nHAp, is a mineral form of calcium apatite and was removed by decalcification during the histological technique. It was verified both by histological observations and histomorphometric measurements. The latter has previously been applied to assess biodegradability of polyhydroxyalkanoate copolymers by Ying et al. [26]. In our study, the BmA decreased from the 2nd to the 4th and from 2nd to the 8th postoperative week, indicating adequate biodegradation of 75/25 w/w nHAp/CS scaffolds.

Zhang et al. [30] used 3D printed polylactic acid/hydroxyapatite (PLA/HA), β-tricalcium phosphate (β-TCP), and partially demineralized bone matrix (DBM) scaffolds. These were evaluated by both micro-computed tomography and histological analysis at 4 and 8 weeks, postoperatively. Compared to β-TCP and DBM, the 3D printed PLA/HA scaffolds showed good biocompatibility and in vitro bioactivity. PLA/HA 3D printed scaffolds also exhibited in vivo mild inflammatory response, improved osteoinductivity, increased NBF, and relatively increased rate of biodegradation [30]. Also, Danilchenko et al. [38] showed that implanted HAp/CS scaffolds in rat calvaria CSD exhibit sufficient biodegradability and concomitant increase in NBF, in terms of histological and histomorphometric analyses, at 10 to 24 days postoperatively. Consequently, the results of our study are comparable to those of methodologically similar bone regeneration studies.

## Conclusions

Our study shows that 75/25 w/w nHAp/CS scaffolds provide an effective space for NBF. As our findings do not necessarily apply to the human alveolar bone, further investigation of 75/25 w/w nHAp/CS scaffolds for bone regeneration in oral surgery-related procedures, i.e., implants’ placement, alveolar ridge augmentation, is required.

## Data Availability

The datasets used and/or analyzed during the current study are available from the corresponding author on reasonable request.
